# Metabolic imaging with FDG-PET and time to progression in patients discontinuing immune-checkpoint inhibition for metastatic melanoma

**DOI:** 10.1186/s40644-022-00449-3

**Published:** 2022-02-05

**Authors:** Justin Ferdinandus, Anne Zaremba, Lisa Zimmer, Lale Umutlu, Robert Seifert, Francesco Barbato, Selma Ugurel, Eleftheria Chorti, Viktor Grünwald, Ken Herrmann, Dirk Schadendorf, Wolfgang Peter Fendler, Elisabeth Livingstone

**Affiliations:** 1 Department of Nuclear Medicine, University of Duisburg-Essen and German Cancer Consortium (DKTK)-University Hospital Essen, Hufelandstr, 55, 45147 Essen, Germany; 2 Department of Dermatology, University of Duisburg-Essen and German Cancer Consortium (DKTK), University Hospital Essen, Hufelandstr, 55, 45147 Essen, Germany; 3 Department of Diagnostic and Interventional Radiology and Neuroradiology, University Hospital Essen, University of Duisburg-Essen, Essen, Germany; 4 Department of Medical Oncology, West German Cancer Center, University Hospital Essen, Essen, Germany

**Keywords:** Melanoma, Immunotherapy, Checkpoint inhibition, PET, FDG, Discontinuation

## Abstract

**Background:**

The optimal duration of immune checkpoint blockade (ICB) therapy is not well established. Active residual disease is considered prohibitive for treatment discontinuation and its detection by diagnostic CT imaging is limited. Here, we set out to determine the potential added value of 2-[18F]fluoro-2-deoxy-D-glucose positron emission tomography (FDG-PET) to identify patients at higher risk of relapse following discontinuation of ICB in advanced melanoma.

**Methods:**

Metastatic melanoma patients who discontinued ICB were identified retrospectively. Eligible patients received FDG-PET and diagnostic CT within four months of ICB discontinuation. We defined morphologic response using RECIST v1.1. Complete metabolic response (CMR) was defined as uptake in tumor lesions below background, whereas any site of residual, FDG-avid disease was rated as non-CMR. The primary endpoint was time to progression (TTP) after therapy discontinuation stratified by morphologic and metabolic imaging response using Kaplan–Meier estimates and log-rank test.

**Results:**

Thiry-eight patients were eligible for this analysis. Median follow-up was 37.3 months since ICB discontinuation. Median TTP in the overall cohort was not reached. A greater proportion of patients were rated as CMR in PET (*n* = 34, 89.5%) as compared to complete response (CR) in CT (*n* = 13, 34.2%). Median TTP was reached in patients with non-CMR (12.7 months, 95%CI 4.4-not reached) but not for patients with CMR (log-rank: *p* < 0.001). All patients with complete response by CT had CMR by PET. In a subset of patients excluding those with complete response by CT, TTP remained significantly different between CMR and non-CMR (log-rank: *p* < 0.001).

**Conclusion:**

Additional FDG-PET at time of discontinuation of ICB therapy helps identify melanoma patients with a low risk of recurrence and favourable prognosis compared to CT imaging alone. Results may have clinical relevance especially for patients with residual tumor burden.

**Supplementary Information:**

The online version contains supplementary material available at 10.1186/s40644-022-00449-3.

## Background

Immune checkpoint blockade (ICB) with programmed cell death protein-1 (PD-1) and/or cytotoxic T lymphocyte antigen-4 (CTLA-4) inhibitors remarkably improved 5-year overall survival (OS) rates in patients with metastatic melanoma [1–4]. In contrast to conventional chemotherapy, ICB can induce high rates of durable responses, even when treatment is discontinued [5]. In the CheckMate 067 trial, 74% patients in the combined anti-PD-1 and anti-CTLA-4 treatment arm who were still alive after 5 years (*n* = 151) had discontinued immunotherapy and had not received subsequent systemic therapy [4]. In the phase 3 KEYNOTE-006 study, sustained efficacy was seen in patients who discontinued therapy according to protocol after 2 years of treatment [3]. OS of patients who stopped treatment with stable disease as best response, however, was inferior to patients with partial or complete response [6].

Optimal duration of ICB treatment in patients with metastatic melanoma has not been defined yet. Most clinical trials evaluating PD-1 and PD-L1 inhibitors for metastatic melanoma did not limit duration of treatment and drugs were to be given until disease progression, unacceptable toxicitiy or withdrawal of consent [7]. Few clinical trials have observed patients who discontinued treatment after responding to therapy, and prospective studies exploring the optimal timing of treatment discontinuation have only recently started recruitment [8].

A current review suggested that discontinuation of ICB can be considered in advanced melanoma patients who show a complete response (CR) for at least six months after discussion of risks and benefits. If partial response (PR) or stable disease (SD) is present and unchanged for at least six months, additional 2-[18F]fluoro-2-deoxy-D-glucose positron emission tomography (FDG-PET) imaging and pathological assessment could identify patients without vital disease who could safely pause immunotherapy [7].

This study investigated the value of FDG-PET to determine the potential added value of FDG-PET to further identify patients with residual morphologic disease and at risk of relapse following discontinuation of immunotherapy in metastatic melanoma.

## Methods

This is a monocentric, retrospective study of metastatic melanoma patients who discontinued immunotherapy. To identify eligible patients, institutional database records were searched for melanoma patients who discontinued immunotherapy for either unacceptable toxicity or durable response (i.e. at least 6 months of tumor control on conventional imaging and decision to discontinue based on multidisciplinary tumorboard) between 2010 and 2020.

Patients who received adjuvant ICB were excluded. Eligible metastatic patients had to have discontinued treatment with CTLA-4 and/or PD-1 inhibitors due to unacceptable toxicity and/or durable response and have received both, FDG-PET and diagnostic CT examinations within 4 months of discontinuation of ICB. Decision for therapy cessation in most cases was discussed due to durable CT-response and additional FDG-PET to detect residual metabolic disease at the time of discontinuation. Patients were not included in this analysis if they had switched or stopped ICB due to progression.

Diagnostic CT (i.e. full dose and contrast enhanced) as part of PET/CT imaging was permitted (*n* = 33). In case of low-dose PET/CT, a post-treatment full-dose CT scan within 6 weeks of discontinuation was required (observed range 6–38 days) (*n* = 5). Morphologic CT response was defined by RECIST v1.1. FDG-PET was performed as per EANM guidelines [9]. In brief, patients received a mean activity of 314 ± 75 MBq FDG and underwent PET following a mean of 67 ± 17 min after injection. CT images were used for attenuation correction. Complete metabolic response (CMR) at the time of discontinuation was defined as uptake in tumor lesions below background levels, using mediastinal blood pool as reference. Most patients had no baseline scan prior to ICB for comparison; any site of residual, FDG-avid disease was therefore rated as non-CMR. In patients with cerebral metastases (*n* = 3) (stage M1d according to American Joint Cancer Committee (AJCC) 8^th^ edition), cranial magnetic resonance imaging (MRI) response was also considered. Following the evaluated FDG-PET and CT, patients received a standardized follow-up consisting of staging examinations comprising CT images of the thorax, abdomen, and MRI of the skull every three months as per clinical routine. Additional imaging examinations were added depending on individual indication. Staging examinations were continued at three-monthly intervals and therapy response was assessed for all patients during and after therapy discontinuation.

The primary endpoint was time to progression (TTP) stratified by morphologic response (i.e. grouped into CR, PR and SD as per RECIST v1.1) using diagnostic CT and cranial MRI in case of cerebral metastasis and metabolic response (i.e. grouped into CMR and non-CMR) using PET. TTP was defined as time from discontinuation until clinical or radiological evidence of tumor progression or recurrence. Survival of subgroups was compared using Kaplan–Meier methods and log-rank comparisons.

As secondary endpoint, we report overall survival (OS) measured from time of therapy start. Chi-squared test was used to compare the rate of recurrence within 12 months between complete responders from morphologic and metabolic imaging. The relative risk (RR) was calculated as the ratio of the probability of relapse within 12 months. Grade 3 or higher toxicities were recorded using Common Terminology Criteria of Adverse Events (CTCAE Version 5.0) definitions. All statistical analyses were performed using R statistics (version 3.4.1, www.r-project.org). The study was approved by the local ethics committee (Reference: 20–9433-BO).

## Results

We identified 38 eligible patients who discontinued ICB. Patient characteristics are outlined in Table 1. Median age at the time of PET scan was 58 years (range 33–86); 22 patients (57.9%) were male. Most patients had received combined immunotherapy (*n* = 24, 63.2%). PD-1 monotherapy was either nivolumab (*n* = 5, 13.2%) or pembrolizumab (*n* = 9, 23.7%). Seven patients (18.4%) had received prior lines of ICB, e.g. as adjuvant therapy. A BRAF mutation was present in 15 patients (39.5%). Median treatment duration was 19.0 months (range 0.7–48.0). Twenty-three out of 38 patients (60.5%) experienced toxicities greater or equal to CTCAE grade 3. Most frequent toxicities were colitis (*n* = 7, 18.4%) and hepatitis (*n* = 6, 15.8%). Detailed information on adverse events in this cohort can be found in the supplement.

**Table 1:** Patient characteristics

**Characteristic**	**Reason for discontinuation**		**Overall** **(*n* = 38)**
	**Durable response (*n* = 27)**	**Toxicity** **(*n* = 11)**	
**Age**			
Median (range)	58 (38 – 81)	52 (33—86)	58 (33—86)
**Sex**			
female	13 (48.1%)	3 (27.3%)	16 (42.1%)
male	14 (51.9%)	8 (72.7%)	22 (57.9%)
**AJCC**			
IIIB	4 (14.8%)	1 (9.1%)	5 (13.2%)
IV M1a	1 (3.7%)	3 (27.3%)	4 (10.5%)
IV M1b	4 (14.8%)	2 (18.2%)	6 (15.8%)
IV M1c	14 (51.9%)	5 (45.5%)	19 (50.0%)
IV M1d	4 (14.8%)	0 (0%)	4 (10.5%)
**BRAF status**			
Mutated	10 (37.0%)	5 (45.5%)	15 (39.5%)
Wildtype	17 (63.0%)	5 (45.5%)	22 (57.9%)
Not reported	0 (0%)	1 (9.1%)	1 (2.6%)
**Regimen**			
Ipilimumab/Nivolumab	16 (59.3%)	8 (72.7%)	24 (63.2%)
Nivolumab	4 (14.8%)	1 (9.1%)	5 (13.2%)
Pembrolizumab	7 (25.9%)	2 (18.2%)	9 (23.7%)
**Duration of immunotherapy**			
Median (range)	24 (8.7 – 48)	1.3 (0.69—22)	19 (0.69—48)
**Prior surgery**	22 (81.5%)	9 (81.8%)	31 (81.6%)
**Prior radiotherapy**	8 (29.6%)	2 (18.2%)	10 (26.3%)
**Prior systemic therapy**			
IFN	8 (29.6%)	1 (9.1%)	9 (23.7%)
Immunotherapy	7 (25.9%)	0 (0%)	7 (25.9%)
Targeted therapy	4 (14.8%)	3 (27.3%)	7 (18.4%)
Chemotherapy	2 (7.4%)	0 (0%)	2 (5.3%)
Other	2 (7.4%)	1 (9.1%)	3 (7.9%)
**Toxicity ≥ grade 3**	12 (44.4%)	11 (100%)	23 (60.5%)
**LDH elevated**^**a**^	3 (11.1%)	2 (18.2%)	5 (13.2%)
**S100 elevated**^**a**^	0 (0%)	3 (27.3%)	3 (7.9%)
**RECIST v1.1 group**^**a**^			
CR	11 (40.7%)	2 (18.2%)	13 (34.2%)
PR	14 (51.9%)	7 (63.6%)	21 (55.3%)
SD	2 (7.4%)	2 (18.2%)	4 (10.5%)

^a^at time of discontinuation

Abbreviations: *AJCC* American Joint Classification of Cancer

Treatment was discontinued due to unacceptable toxicity in 11 patients (28.9%) and due to durable response in 27 patients (71.1%). Median time to therapy discontinuation for patients ceasing therapy for durable response was 24.0 months (range 8.7–48.0) from ICB start and 1.3 months (range 0.7–22.0) in patients who discontinued due to unacceptable toxicity.

FDG-PET was performed within four months of treatment cessation (median 0.3, range -3.7–4.0). PET identified a greater proportion of patients with complete response compared to RECIST in CT. According to CT, four patients (10.5%), had stable disease (SD), 21 (55.3%) had a PR, and 13 (34.2%) had a CR at the time of treatment discontinuation CT. Based on FDG-PET, 34 (89.5%) patients had CMR and four (10.5%) patients had non-CMR. Of 34 patients with CMR in PET, 13/34 (38.2%), 18/34 (52.9%) and 3/34 (8.8%) had CR, PR and SD in CT, respectively. Of four patients with non-CMR, 3/4 (75.0%) and 1/4 (25.0%) were classified as PR and SD by CT. In total, 21 patients with residual, non-progressive disease (i.e. PR or SD) in CT were identified with CMR in PET. Table 2 shows a comparison of PET and CT responses.

**Table 2:** Comparison of PET and CT responses at time of discontinuation

	**CMR** **(*n* = 34)**	**Non-CMR** **(*n* = 4)**
RECIST v1.1 response*		
CR (*n* = 13)	13 (38.2%)	0 (0%)
PR (*n* = 21)	18 (52.9%)	3 (75.0%)
SD (*n* = 4)	3 (8.8%)	1 (25.0%)

Abbreviations: *CR* complete response, *CMR*, complete morphological response, *PR* partial response, *SD* stable disease

**Fig. 1:**
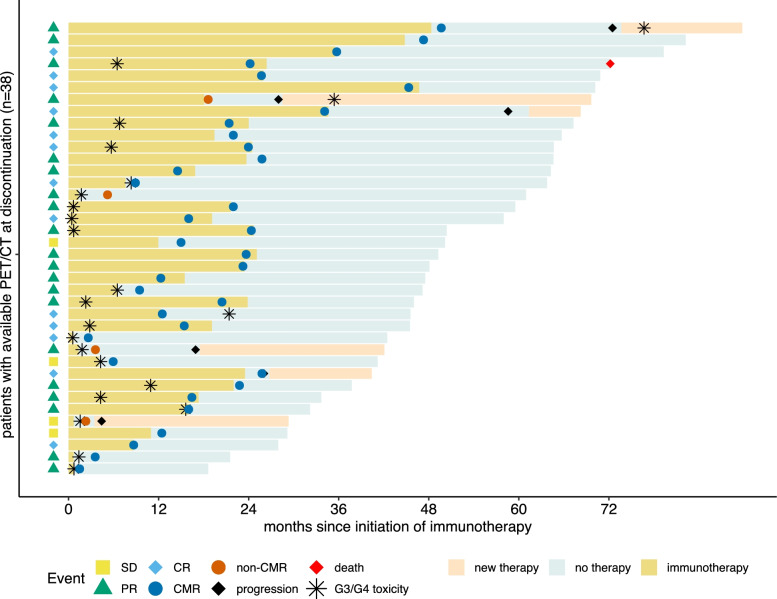
Swimmers plot of PET-patient events following initiation of immunotherapy. * RECIST Responses at time of discontinuation; Symbols may be placed on top of each other

Median TTP of the total cohort was not reached. At a median follow-up time of 37.3 months since immunotherapy discontinuation, disease relapse had occurred in six patients (Additional file table 3), four had ceased immunotherapy for durable response and two for unacceptable toxicity. Three out of four (75.0%) patients with non-CMR at time of discontinuation had progression, and threeout of thirty-four (8.8%) patients with CMR. Of the patients with relapse, 2/6 (33.3%), 3/6 (50.0%) and 1/6 (16.7%) patients had CR, PR and SD according to CT-assessed RECIST at the time of discontinuation, respectively. According to PET, 3/6 (50.0%) patient with relapse after discontinuation had been rated CMR at the time of discontinuation and 3/6 (50.0%) patients had been rated non-CMR. Fig. **1** illustrates patients’ events since start of ICB.

All thirty eight patients had > 12 months of follow-up and were thus available for analysis of relative risk for progression within twelve months after discontinuation. Patients without complete response in metabolic imaging had higher relative risk (RR) of recurrence (RR 8.3, 95%CI 2.4–27.9); Chi-square: *p* = 0.008). Results were not significant for morphologic imaging (RR 1.1, 95%CI 0.2- 5.1; Chi-square: *p* = 0.9).

**Fig. 2:**
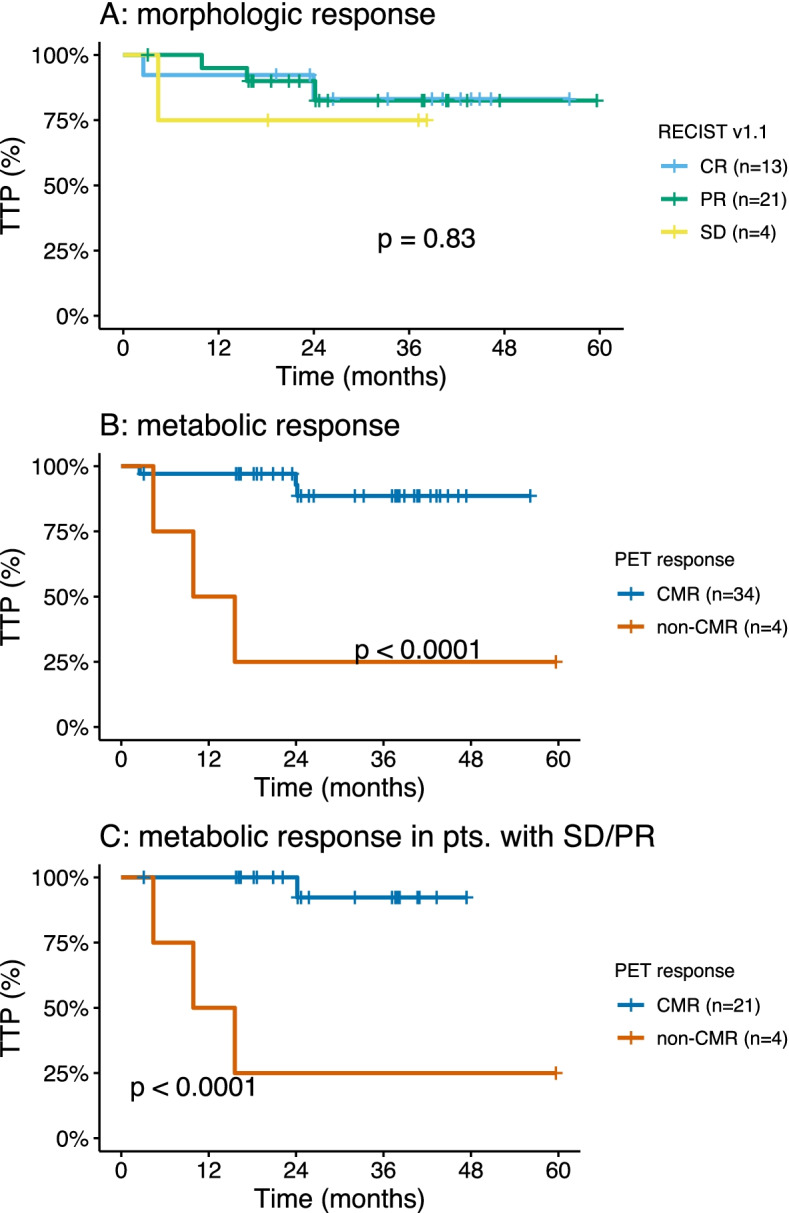
Time to progression after ending immunotherapy. CMR = complete metabolic response; PD = progressive disease; SD = stable disease; PR = partial response; CR = complete response

Median TTP was not reached in any RECIST subgroup (Fig. 2a). Patients with non-CMR had a significantly shorter TTP compared to CMR patients (12.7 months, 95%CI 4.4-NR, vs not reached, *p* < 0.0001, Fig. 2B).

In patients with residual, non-progressive disease (i.e. PR and SD) identified by CT (*n* = 25), further classification was achieved by metabolic imaging. From these, 21/25 (84.0%) patients had CMR identified by PET, which was associated with longer TTP compared to non-CMR (12.7 months, 95%CI 4.4-NR, vs not reached, *p* = 0.0007, Fig. 2C). Three of 34 (8.8%) patients with CMR at therapy discontinuation received subsequent systemic treatment with either pembrolizumab therapy, adjuvant nivolumab monotherapy after complete resection of recurrence, or combinational therapy of ipilimumab plus nivolumab. Recurrence occurred 2.5, 24.2 and 24.0 months after therapy discontinuation, respectively (Additional file Table 3), all other patients with CMR had no recurrence until data cut and did not receive subsequent therapy. Three patients with non-CMR received subsequent systemic therapies following disease progression, hereof two patients with PD-1 monotherapy and one patient underwent surgery. Response to subsequent systemic therapy was CR in one patient (16.7%), PR in one patient (16.7%) and SD in another (16.7%). Two patients with CR at time of discontinuation have received subsequent therapy. Detailed outcomes of patients with relapse are listed in Additional file table 3.

Median OS was not reached. One patient died with unknown cause of death. Median survival of subgroups was also not reached. Survival curves for overal survivall can be found in the supplement.

## Discussion

Durable responses and ongoing remissions even after discontinuation of ICB [3, 4] evoke hope of cure in both patients and physicians. Currently, however, no accurate instrument is available that identifies patients who do not require further maintenance treatment. Although toxicities usually occur early after starting ICB, the possibility of late-emergent toxicity as well as time burden for both the patient and the healthcare system give a strong reason to discuss the required treatment duration with ICB [8]. Biomarkers that reliably differentiate between patients who require additional treatment and those who can safely stop treatment are urgently needed.

Three modalities are available to exclude residual disease to allow safe treatment discontinuation: blood-based biomarkers, histology, and imaging. Among blood-based biomarkers, circulating tumor DNA (ctDNA) has efficacy in predicting and monitoring response of melanoma to immunotherapy [10–12]. To date however, liquid biopsies are not routinely used for the detection of residual disease following.

No published study has systematically obtained histologic specimen in patients following palliative immunotherapy of melanoma, but some knowledge can be derived from neoadjuvant ICB in other solid tumors [13]. However, pathological assessment is not an option for patients with metastatic disease at multiple sites.

Metabolic imaging with FDG-PET complements morphologic assessments, especially with predominant hybrid PET/CT or PET/MRI scanners available. FDG-PET scans are highly effective in staging of advanced melanoma [14] and have been demonstrated to be able to detect early metabolic responses that are predictive of survival [15–17] In routine practice, however, regular (re-)staging is performed by means of CT and MRI examinations as PET scans are often not reimbursed. Notably, the socioeconomic burden of ICB beyond clinical benefit would greatly outweigh the cost of additional PET scans for this cohort, which supports the rationale to test prospectively PET-based treatment discontinuation [8].

To date, most publications on FDG-PET response assessment of ICB in malignant melanoma have focused on predicting responses early in the course of treatment to identify non-responding patients. Iravani et al. found that PET response after a median of 2.4 months of combination ICB was predictive of survival. Additionally, inflammatory findings in PET were also capable of detecting immune-related adverse events even before clinical onset [15]. Ito et al. evaluated FDG-PET for monitoring response to ipilimumab and found FDG-PET response to be a strong predictor of overall survival but identified pseudo-progression in non-target lesions as a potential pitfall [16].

Kong et al. investigated the added value of FDG-PET to detect residual metabolic disease in 27 melanoma patients receiving anti-PD-1 therapy [18]. In their study, five patients with CMR stopped treatment and did not develop a recurrence within a 6–10 months follow-up period [18]. We here present outcomes of 38 patients discontinuing ICB of which 34 had a CMR. After a median follow-up of 37.3 months, three (8.8%) of these patients had disease relapse after > 24 months of therapy discontinuation, with completely resectable in one, complete response to reinitiation of pembrolizumab in another, suggesting that for patients with CMR the risk of relapse is low and relapse might be treatable. This demonstrates that favourable metabolic response on FDG-PET can further select patients with durable tumor control to ICB and underlines its value to guide treatment discontinuation. In this regard, Schank et al. found that metabolic responsiveness predicted disease progression in patients without progression at therapy discontinuation [19].

We observed a low rate of relapse in patients with morphologic complete response and no patient with morphologic complete response had residual metabolic disease. Therefore, additional FDG-PET to assess risk for relapse might be of greatest value in patients with durable SD and PR seen in CT. More than half of all patients in this study achieved only PR or SD according to RECIST v1.1 but were complete metabolic responders by PET. Similar results had been observed for patients under continued immunotherapy. Following 12 months of immunotherapy, 68% of melanoma patients with PR in CT had CMR in FDG-PET, which was associated with longer progression-free survival when compared to patients with non-CMR [20].

The ongoing phase II PET-Stop trial (NCT04462406) investigates the feasibility of PET-guided discontinuation of anti PD-1 therapy. To be eligible for active surveillance, patients will either have to have a negative PET-scan or a negative biopsy of PET-positive regions to rule out residual tumor after 52 weeks of immunotherapy. However, results are not expected before 2026. In the meantime, discontinuation will remain an individual approach. Our study provides a rationale for FDG-PET in multiple settings arising during ICB. These include e.g. [1] prior to per-protocol discontinuation of ICB, [2] when toxicity limits therapy continuation, [3] if patients wish to cease systemic therapy, to guide decision making, especially in patients with residual disease visible in CT.

Although the present study benefits from long follow-up with regular imaging (typically every three months), it is limited by the retrospective assessment and a small sample size and does not serve as definitive evidence. Furthermore, we did not perform systematic biopsies in these patients and therefore, non-CMR was often not confirmed. Despite this, high rates of relapse following non-CMR scans support the hypothesis that these patients had residual disease.

Finally, there is need for biomarker-driven, randomized trials in advanced melanoma patients to determine safety, quality of life benefit, socioeconomic impact and (most-importantly) non-inferiority of discontinuation especially in presence of CMR.

## Conclusion

Compared to morphologic imaging with CT, metabolic imaging with FDG-PET identified a greater proportion of patients with complete responses, who were associated with a low rate of relapse after treatment discontinuation. PET-CT may more accurately identify patients at risk who require closer surveillance or further treatment. Prospective studies investigating PET-based treatment discontinuation are warranted.

## Supplementary Information

**Additional file 1: Table 1. **Type and frequency of G3/G4 toxicity.

**Additional file 2: Table 2. **Relative risk of Progression within 12 months after discontinuation.

**Additional file 3: Table 3. **Outcomes of patients with relapse.

**Additional file 4: Table 4. **Response by reason of discontinuation.

**Additional file 5: Figure 1.** Overall survival from therapy start.

**Additional file 6: Figure 2.** Comparison of CT- and PET-imaging responses.

## Data Availability

Data are available on reasonable request.
