# Supplementary material for: Metabolic imaging with FDG-PET and time to progression in patients discontinuing immune-checkpoint inhibition for metastatic melanoma
Source: Cancer Imaging. 2022 Feb 5;22:11. doi: 10.1186/s40644-022-00449-3 (PMC8817553; doi:10.1186/s40644-022-00449-3)
Supplement: Supplementary file 1 — Additional file 1: Table 1. Type and frequency of G3/G4 toxicity. [file 40644_2022_449_MOESM1_ESM.docx]

**Supplemental table 1:** Type and frequency of G3/G4 toxicity

| Adverse event | Frequency (%) | ceased ICB due to toxicity |
| --- | --- | --- |
| colitis | 7 (18.4%) | 3/7 (42.9%) |
| hepatitis | 6 (15.8%) | 3/6 (50.0%) |
| hypophysitis | 3 (7.9%) | 1/3 (33.3%) |
| pneumonitis | 2 (5.3%) | 0/2 (0.0%) |
| arthralgia | 1 (2.6%) | 1/1 (100%) |
| CK-Elevation | 1 (2.6%) | 1/1 (100%) |
| myasthenia | 1 (2.6%) | 0/1 (100%) |
| nephritis | 1 (2.6%) | 1/1 (100%) |
| pancreatitis | 1 (2.6%) | 1/1 (100%) |

Abbreviations: CK: creatinine kinase; ICB: immune checkpoint blockade; SIRS = Systemic inflammatory response syndrome
