# Supplementary material for: Metabolic imaging with FDG-PET and time to progression in patients discontinuing immune-checkpoint inhibition for metastatic melanoma
Source: Cancer Imaging. 2022 Feb 5;22:11. doi: 10.1186/s40644-022-00449-3 (PMC8817553; doi:10.1186/s40644-022-00449-3)
Supplement: Supplementary file 2 — Additional file 2: Table 2. Relative risk of Progression within 12 months after discontinuation. [file 40644_2022_449_MOESM2_ESM.docx]

**Supplemental Table 2:** Relative risk of Progression within 12 months after discontinuation

| Comparison | Rate of Progression (%) | RR (95%CI) | P  *Chi-Square* |
| --- | --- | --- | --- |
| Morphologic CR (CR vs. non-CR) | 4/25 (16.0%) vs. 2/13 (15.4%) | 1.0 (0.2- 4.9) | 0.96 |
| Metabolic CR (CMR vs. non-CMR) | 3/34 (8.8%) vs. 3/4 (75.0 %) | 8.5 (2.5-28.8) | 0.0006 |

Abbreviations: CI: confidence interval; CR: complete response; CMR: complete morphological response, RR: relative risk: partial response, SD: stable disease; PD: progressive disease
