# Supplementary material for: Metabolic imaging with FDG-PET and time to progression in patients discontinuing immune-checkpoint inhibition for metastatic melanoma
Source: Cancer Imaging. 2022 Feb 5;22:11. doi: 10.1186/s40644-022-00449-3 (PMC8817553; doi:10.1186/s40644-022-00449-3)
Supplement: Supplementary file 3 — Additional file 3: Table 3. Outcomes of patients with relapse. [file 40644_2022_449_MOESM3_ESM.docx]

**Supplemental Table 3:** Outcomes of patients with relapse

| Patient | AJCC stage | BRAF status | Immunotherapeutic regimen | Duration of Immunotherapy (months) | Reason for discontinuation | CT Response at discontinuation | PET Response at discontinuation | Time until relapse (months) | New therapy | Best Response to new therapy | Death |
| --- | --- | --- | --- | --- | --- | --- | --- | --- | --- | --- | --- |
| 1 | 4 | V600E | PEMBRO | 23.5 | CR/CMR | CR | CMR | 2.5 | PEMBRO | CR | NO |
| 2 | 4 | V600G | IPI/NIVO | 18.1 | CR/CMR | PR | Non-CMR | 9.9 | NIVO | PD | NO |
| 3 | 4 | wt | IPI/NIVO | 0.7 | toxicity | SD | Non-CMR | 4.4 | Metastasectomy | NA | NO |
| 4 | 4 | V600E | PEMBRO | 48.3 | CR/CMR | PR | CMR | 24.2 | Surgery + NIVO | NA | NO |
| 5 | 4 | wt | IPI/NIVO | 1.4 | toxicity | PR | Non-CMR | 15.6 | PEMBRO | SD | NO |
| 6 | 4 | wt | NIVO | 34.6 | CR/CMR | CR | CMR | 24.0 | IPI/NIVO | PR | NO |

Abbreviations: AJCC: American Joint Classification of Cancer; CMR: complete morphological response, IPI: Ipilimumab, NIVO: Nivolumab, PEMBRO: Pembrolizumab, PD: progressive disease; PR partial response; SD: stable disease; wt: wild type
