# Supplementary material for: Metabolic imaging with FDG-PET and time to progression in patients discontinuing immune-checkpoint inhibition for metastatic melanoma
Source: Cancer Imaging. 2022 Feb 5;22:11. doi: 10.1186/s40644-022-00449-3 (PMC8817553; doi:10.1186/s40644-022-00449-3)
Supplement: Supplementary file 4 — Additional file 4: Table 4. Response by reason of discontinuation. [file 40644_2022_449_MOESM4_ESM.docx]

**Supplemental Table 4:** Response by reason of discontinuation

|  | Durable response | | | Toxicity | | |
| --- | --- | --- | --- | --- | --- | --- |
|  | **CR (n=11)** | **PR (n=14)** | **SD (n=2)** | **CR (n=2)** | **PR (n=7)** | **SD (n=2)** |
| CMR | 11 (100%) | 13 (92.9%) | 2 (100%) | 2 (100%) | 5 (71.4%) | 1 (50.0%) |
| non-CMR | 0 (0%) | 1 (7.1%) | 0 (0%) | 0 (0%) | 2 (28.6%) | 1 (50.0%) |

CR: complete response; CMR: complete morphological response, PR: partial response, SD: stable disease; PD: progressive disease
