# Supplementary material for: Metabolic imaging with FDG-PET and time to progression in patients discontinuing immune-checkpoint inhibition for metastatic melanoma
Source: Cancer Imaging. 2022 Feb 5;22:11. doi: 10.1186/s40644-022-00449-3 (PMC8817553; doi:10.1186/s40644-022-00449-3)
Supplement: Supplementary file 5 — Additional file 5: Figure 1. Overall survival from therapy start. [file 40644_2022_449_MOESM5_ESM.docx]

**Supplemental Figure 1**: Overall survival from therapy start
