# Supplementary figures and images for: Metabolic imaging with FDG-PET and time to progression in patients discontinuing immune-checkpoint inhibition for metastatic melanoma
Source: Cancer Imaging. 2022 Feb 5;22:11. doi: 10.1186/s40644-022-00449-3 (PMC8817553; doi:10.1186/s40644-022-00449-3)

**Supplemental Figure 2**: Comparison of CT- and PET-imaging responses


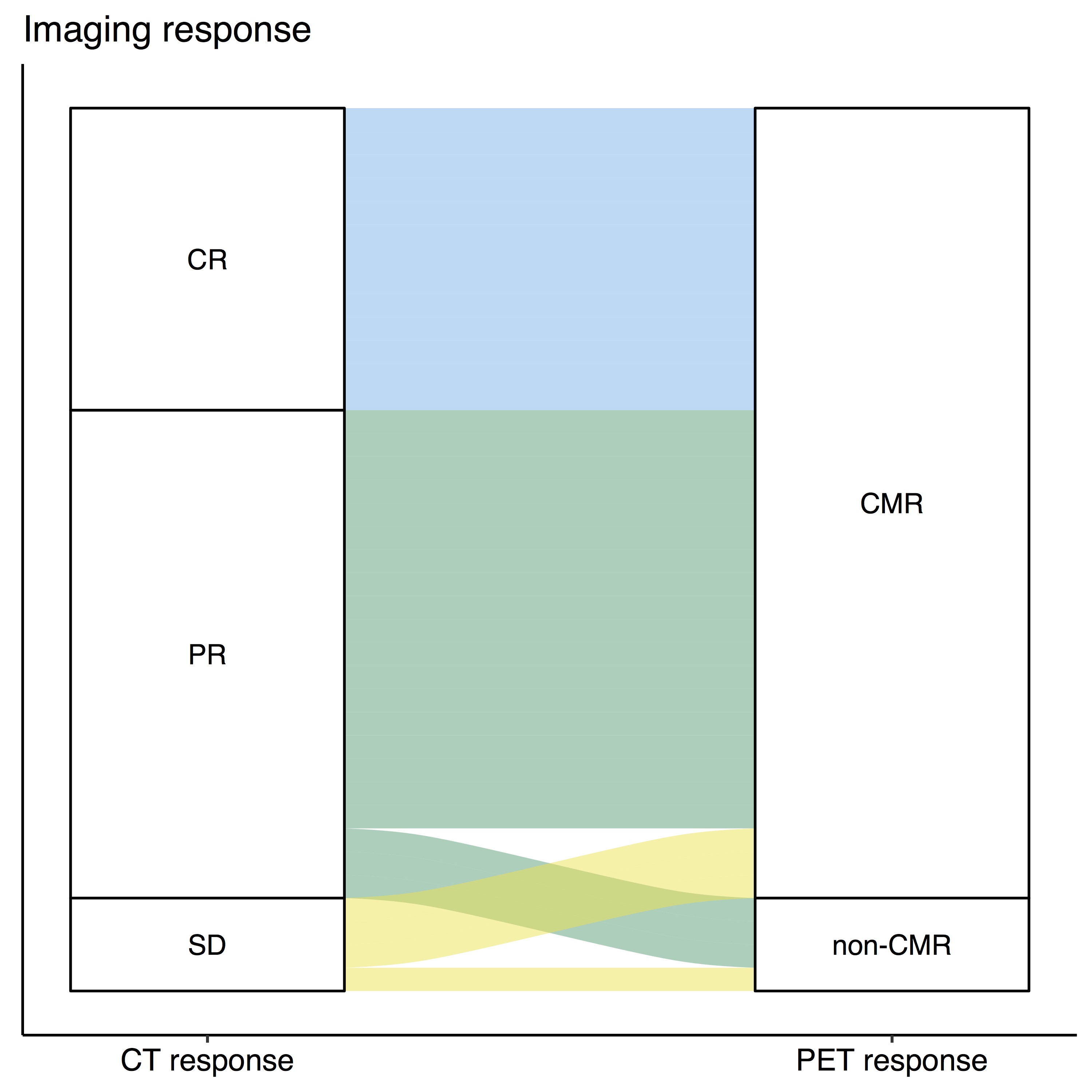

Supplement: Supplementary file 6 — Additional file 6: Figure 2. Comparison of CT- and PET-imaging responses. [file 40644_2022_449_MOESM6_ESM.docx]
